# Association between standardized vitamin 25(OH)D and dyslipidemia: a community-based study in Riyadh, Saudi Arabia

**DOI:** 10.1186/s12199-019-0841-5

**Published:** 2020-01-15

**Authors:** AlJohara M AlQuaiz, Ambreen Kazi, Randa M. Youssef, Norah Alshehri, Shatha Ahmed Alduraywish

**Affiliations:** 1 Princess Nora Bent Abdullah Chair for Women’s Health Research, King Saud University, PO Box 231831, Riyadh, 11321 Kingdom of Saudi Arabia; 2 King Saud University-Medical College -Department of Family and Community Medicine, Riyadh, Kingdom of Saudi Arabia

**Keywords:** Vitamin D deficiency, HDL cholesterol, Triglycerides, Gender differences, Saudi Arabia

## Abstract

**Background:**

Vitamin D deficiency associated with dyslipidemia can contribute towards cardiovascular diseases. Previous studies have found that Saudi Arabia has a high burden of vitamin D deficiency and cardiovascular disease risk factors. We aimed to explore the relationship between vitamin D deficiency and dyslipidemia, including total cholesterol, low-density lipids, high-density lipids (HDL), and triglycerides (TG) in apparently healthy Saudi male and female participants aged 30–75 years.

**Methods:**

A cross-sectional study was conducted on 1717 apparently healthy Saudi participants from 18 primary health care centers in Riyadh. Data collectors conducted the interviews, took anthropometric measurements, and collected the blood samples. Serum 25-hydroxyvitamin vitamin D (25(OH)D) levels were measured using an electrochemiluminescence assay method. Lipid panel was measured by a fully automated analyzer using enzymatic methods.

**Results:**

Multivariable logistic regression analysis revealed that the adjusted odds ratio (OR_*A*_) of low level of HDL cholesterol in association with 25(OH)D deficiency was 2.1 times higher in males (OR_*A*_ = 2.1; 95% CI = 1.1, 3.9) and 1.3 times higher in females (OR_*A*_ = 1.3; 95% CI = 0.9, 1.9). A significant excess odds ratio of high levels of TG in association with 25(OH) D deficiency was observed in females (OR_*A*_ = 3.0; 95% CI = 1.1, 7.9) but not in males.

**Conclusion:**

Vitamin D deficiency is highly prevalent in Saudi Arabia. Low levels of HDL cholesterol in men and high TG levels in women are associated with vitamin D deficiency. The results emphasize the importance of treating vitamin D deficiency in the general population.

## Introduction

Vitamin 25(OH) D deficiency is defined as a serum 25-hydroxyvitamin vitamin D (25(OH)D) level of less than 50 nmol/L (20 ng/mL) [1]. Vitamin D deficiency is common worldwide and has become an important public health problem in many countries [2]. In the Kingdom of Saudi Arabia (KSA), vitamin D deficiency has been identified in both genders and all age groups [3–6]. A review study by Daghri et al. reported that the overall prevalence of vitamin D deficiency (< 50 nmol/L) was around 81% [5]. Recently, a population-based study conducted by AlQuaiz et al. found that 72% of males and 64% of females aged 30–75 years had vitamin D levels of < 50 nmol/L [6].

Recently, a growing body of evidence has suggested that, in addition to its role in bone health, vitamin D has various other functions, such as reducing insulin resistance in pregnant women [7], modulating immune function [8], suppressing the renin–angiotensin system [9], acting as an anti-inflammatory [10], and preventing musculoskeletal disorders [11]. It is suggested that vitamin D receptors exist on different cell types, including cardiomyocytes, vascular endothelial cells, and immune cells [12]. Studies have found that vitamin D deficiency may increase the risk of hypertension, left ventricular hypertrophy, chronic vascular inflammation, diabetes and metabolic syndrome, and congestive heart failure [1, 2, 5].

Epidemiological studies from elsewhere have shown that an association exists between low vitamin D levels, dyslipidemia, and cardiovascular diseases (CVD) [13, 14]. A meta-analysis based on the results of 16 studies assessed the dose–response association between circulating vitamin 25(OH)D and the risk of CVDs [15]. The findings of this meta-analysis showed that the risk of CVD marginally increases with decreasing vitamin D concentration below 60 nmol/L (pooled relative risk = 1.03 (95% CI = 1.00–1.06) per 25 nmol/L decrement in 25(OH)D vitamin [15].

Lately, studies from Saudi Arabia have reported a significant increase in the incidence of CVD and the associated risk factors [16, 17]. A review study on CVD risk factors in the Middle East and North Africa (MENA) region reported a wide variation in the prevalence and factors associated with CVD [16, 17]. A study conducted on 500 participants from KSA found that 50% of these individuals had more than three CVD risk factors, with dyslipidemia being the most common (68.6%) [17]. Recent studies from Saudi Arabia focused on vitamin D deficiency and the effect of vitamin D supplementation among diabetic patients. The rates of vitamin D deficiency are significantly higher among diabetic patients of type 1 [18] and type 2 [19] compared to healthy controls. Intervention studies of vitamin D supplementation among Saudi diabetic patients yielded inconsistent findings. Some studies reported that vitamin D supplementation is associated with an improvement in glycemic control [20] and lipid profile [21] even at below optimum level of 25(OH)D [20] while others reported the absence of improvement in glycemic control [22, 23] or in lipid profile [22]. Moreover, to prove any true association, it is important to include standardized vitamin D results to avoid any discrepancies due to methodological differences [24].

Despite the high rate of vitamin D deficiency among healthy population in Saudi Arabia, none of the studies addressed its association with the lipid profile. This study will fill the gap of knowledge by investigating the relation between serum levels of 25(OH)D and lipid profile among a representative national sample of the Saudi population. The objective of this study was to determine the association between standardized 25(OH) vitamin D levels and lipid profiles in healthy Saudi males and females in Riyadh, Saudi Arabia.

## Methods

The data was collected from December 2014 to August 2015 through a large cross-sectional survey, namely, Women in Saudi Arabia Health Examination Survey, (WISHES project). Saudi, male and female participants aged 30–75 years were recruited from 18 randomly selected (https://www.random.org/) primary health care centers (PHCCs) from the five administrative regions of Riyadh City (north, east, west, south, and central).

Initially, 2997 Saudi adults (males = 968, females = 2029) aged between 30 and 75 years were included in the “WISHES” project. The sample size and sampling methodology of the WISHES project are detailed elsewhere [6]. In this study, we excluded participants suffering from hypercholesterolemia and on lipid-lowering drugs (*n* = 690) as well as those suffering from diabetes mellitus (*n* = 679), thyroid gland disorders (*n* = 220), and liver or renal disease (*n* = 33). Participants diagnosed with and being treated for osteoporosis (*n* = 261) and those with a history of CVD (*n* = 66) were also excluded. The final analysis included a total of 1717 healthy participants, 1064 females and 653 males.

### Research instruments and data collection procedures

An interview-based data collection approach was followed by using a structured questionnaire, followed by blood pressure and anthropometric measurements, and blood sample collection. Detailed interviews were conducted to obtain information about socio-demographic characteristics, smoking history, medical history, physical activity, sun exposure, vitamin D supplementation, and reproductive history (from females only). The study protocol was approved by the Institutional Review Board, King Saud University (E-12-658), and the Institutional Review Board of the Ministry of Health, Saudi Arabia (IRB ID MOH0151). Written informed consent was obtained from participants after the purpose of the study was explained clearly, with emphasis on voluntary participation, anonymity, and confidentiality.

### Physical Activity questionnaire

We used the validated International Physical Activity questionnaire (IPAQ, short form) [25]. The items in the short IPAQ form are structured to provide separate scores for walking, moderate-intensity and vigorous-intensity activity. MET minutes/week (metabolic equivalents minutes/week) were calculated based on the duration of PA (in minutes) with number of days (per week) multiplied with pre-assigned values of 2.2, 4.0, and 8.0 for walking, moderate-intensity activities, and vigorous-intensity activities, respectively. Continuous scores were converted into the low, moderate, and high physical activity categories according to the scoring guidelines [25]. Sitting time, which is considered as an indicator of time spent in sedentary activity, was calculated as a continuous variable based on the average time spent sitting on a particular week day (both at work and at home).

### Blood pressure and anthropometric measurements

Two blood pressure readings were taken in sitting position according to the instruction’s manual using the oscillometric method (Omron-5 SeriesTM Blood Pressure Monitor Model BP742—China 2010). The average of both readings was computed for each participant. Anthropometric measurements followed the standard protocol and included weight and height, which were measured using an electronic scale (Secca 220—Hamburg, Germany, 2009) and stadiometer, respectively [26]. Body mass index (BMI) was calculated as weight in kilograms divided by height in meters squared. Waist circumference (WC) was measured at the mid-point between the lowest rib and top of the hip bone (iliac crest) [27].

### Collection of blood samples

Random samples of non-fasting venous blood were collected in two different vials (one with a yellow cap and the other with a purple cap). Venous blood (5 cc) was collected in the yellow-capped tube to analyze basic biochemistry (cholesterol, low-density lipoprotein (LDL), high-density lipoprotein (HDL) and triglycerides (TG)), and another 5 cc was collected in the purple-capped tube for vitamin 25(OH)D analysis. A 22- or 23-gauge needle was used along with a sample adaptor to fill the vials. Both vials were placed in a labeled plastic bag and then refrigerated at a temperature of -2 to-8 ^o^C. The samples were transferred to a storage box (with a maintained temperature) and then transported to King Khalid University Hospital for laboratory analysis.

### Level of vitamin D

Initially, serum 25(OH)D levels were measured using electrochemiluminescence (ECLIA immunoassay, Modular Analytics E170, Roche Diagnostics GmbH, Mannheim, Germany) technique. The intra-assay coefficient of variation, defined as the variation between replicate samples of a single ELISA run, was 6.8%, while the inter-assay coefficient, defined as the variation between separate runs of the same ELISA (e.g., the same assay run on different days with the same samples and standards/controls using the same conditions, instruments, etc.) was 13.1%.

The assay was followed by standardizing vitamin 25(OH)D levels, which was conducted in a laboratory that participates in the Vitamin D External Quality Assessment Scheme (DEQAS) utilizing the automated Roche Elecsys Cobas e411 analyzer (Roche Diagnostics, GmbH, Mannheim, Germany) by means of an electrochemiluminescence immunoassay [28]. The total 25(OH)D values were corrected using a linear regression equation derived by establishing the relationship between initial measured total 25(OH)D values and the DEQAS total target values for five of the DEQAS samples. Following this, 200 stored serum samples were selected for re-measurement from the sorted original 25(OH)D values in the complete data set. The results were correlated with the initial 25(OH)D values to develop a mathematical model that enabled us to convert initial vitamin D values to the true ones. Further details and the correlation graphs between the original and true values are available in a prior publication [28]. A cutoff level of < 50 nmol, as recommended by the Endocrine Society, was utilized to identify vitamin D deficiency, whereas vitamin D levels of > 50 nmol (insufficiency and above) were classified as normal [29].

### Lipid levels

Serum levels of total cholesterol, LDL, HDL, and TG were measured in millimoles per liter by a fully automated analyzer (Siemens Dimension RxL, Germany) using enzymatic methods. The intra-assay and inter-assay coefficients of variation were respectively 0.84 and 1.30 for total cholesterol, 1.9 and 2.1 for HDL, and 0.4 and 1.0 for TG. LDL was calculated by using the Friedewald equation (LDL-C = TC − HDL-C − TG/5) [30]. The hospital’s cutoff values to define dyslipidemias were as follows: cholesterol > 5.20 mmol/L, HDL in males < 1.03 mmol/L, HDL in females < 1.29 mmol/L, LDL > 3.36 mmol/L, and triglycerides > 1.48 mmol/L.

### Statistical analysis

The data were analyzed using the Statistical Package for the Social Sciences (IBM SPSS statistics version 21.0). Data were summarized using the number and percentage, as well as the mean, standard deviation, and the 95% confidence interval (95% CI) of the mean. Student’s *t* test for independent samples and the chi-square test were used to determine the significance of the variables in relation to participants’ gender and vitamin 25(OH)D status. Pearson’s correlation coefficient was calculated to measure the association between continuous variables.

Age was analyzed after dividing into three categories, namely 30–44, 45–59, and 60–75 years. Levels of physical activity were measured, with high physical activity defined as “at least 3 days of activity achieving a minimum total physical activity of at least 1500 MET minutes/week” and a moderate level of activity defined as “5 or more days of 30 minutes moderate-intensity activity.” Those not meeting the requirements for high or moderate levels of activity were categorized as low physical activity. Univariable and multivariable logistic regression analyses were used to estimate the association between vitamin 25(OH)D levels (dependent variable) and lipid profiles (independent variable), including total cholesterol, HDL, LDL, and triglycerides. The results of logistic regression analyses were expressed as the odds ratio and the associated 95% confidence interval (CI) of unadjusted (OR) and adjusted (OR_*A*_) values for participants’ age, smoking status, body mass index, physical activity, and vitamin D supplementation. Statistical significance was determined for two-tailed tests at the 5% level.

## Results

The mean level of standardized 25(OH)D concentration among participants was 33.3 ± 19.1 nmol/L, and vitamin D deficiency was ascertained in 83.3% of them. Compared with females, the males had a significantly lower mean levels of 25(OH)D (31.7 ± 15.7 nmol/L in comparison to 34.3 ± 20.8 nmol/L, *P* < 0.01), and a significantly higher proportion of males were suffering from vitamin D deficiency (88.8% compared with 79.9%, *P* < 0.01).

Table 1 is showing the significant difference in the mean (± SD) values for age, BMI, and lipid profile (HDL, LDL, and TG) between the males and females. The HDL levels were higher in females (1.1 ± 0.3 vs 1.4 ± 0.4), whereas TG were higher in the males (1.7 ± 1.1 vs 1.1 ± 0.7) (Table 1).

**Table 1:** Mean (±SD) values for lipid panel in healthy Saudi male and female participants in Riyadh, Saudi Arabia

Variables	All (*N* = 1717)	Males (*n* = 653)	Females (*n* = 1064)	*P* value
Age (in years)	39.5 ± 9.0	40.1 ± 10.2	39.1 ± 8.3	0.02
Body mass index (kg/m^2^)	30.0 ± 6.3	29.4 ± 6.4	30.4 ± 6.2	0.002
HDL-C (mmol/L)	1.3 ± 0.4	1.1 ± 0.3	1.4 ± 0.4	< 0.001
Triglycerides (mmol/L)	1.3 ± 0.9	1.7 ± 1.1	1.1 ± 0.7	< 0.001
Total cholesterol (mmol/L)	5.0 ± 0.9	5.0 ± 1.1	4.9 ± 0.9	0.25
LDL-C (mmol/L)	3.1 ± 0.8	3.1 ± 0.9	3.0 ± 0.7	0.001

Table 2 presents the frequency and the bivariate analysis with 95% CI for 963 and 1064 healthy Saudi males and females, respectively. Majority of the male participants were young adults, educated, married, and working (Table 2). The males were slightly older in age than the females (40.1 ± 10.2 vs 39.1 ± 8.3).Young males and females (30-45 years) had a significant percentage with low vitamin D values. The males and females in age category 46–70 years showed inverse results, with less number in vitamin D deficiency category (Table 2). Surprisingly, the military (12.6% vs 8.2%) and skilled worker (69% vs 66%) occupations in males had higher percentage in the deficient group than in the normal vitamin D group. The high-income group showed marginally significant association with low vitamin D levels (1.5, 95% CI 0.9, 2.8). Males not taking vitamin D supplements had higher odds for deficiency in comparison to those taking vitamin D supplements (5.7, 95% CI 2.4, 13.6).

**Table 2:** Bivariate analysis showing unadjusted odds ratio and 95% CI between socio-demographic and lifestyle factors with low vitamin D in Saudi male and female participants in Riyadh, Saudi Arabia

	Males (*N* = 653)			Females (*N* = 1064)		
Variable	25(OH)D < 50 nmol/L (*n* = 580)	25(OH)D ≥ 50 nmol/L (*n* = 73)	OR (95% CI)	25(OH)D < 50 nmol/L (*n* = 850)	25(OH)D ≥ 50 nmol/L (*n* = 214)	OR (95% CI)
Age in year categories						
30–45	415 (71.6)	45 (61.6)	1.0	637 (74.9)	144 (67.3)	1.0
46–60	134 (23.1)	21 (28.8)	0.7 ( 0.4,1.2)	202 (23.8)	63 (29.4)	0.7 (0.5, 1.0)
61–75	31 (5.3)	7 (9.6)	0.5 (0.2, 1.2)	11 (1.3)	7 (3.3)	*0.3 (0.1, 0.9)*
Level of education						
Graduate and above	461 (79.5)	59 (80.8)	1.0	375 (44.1)	84 (39.3)	1.0
Intermediate and below	119 (20.5)	14 (19.2)	0.9 (0.5, 1.7)	475 (55.9)	130 (60.7)	0.8 (0.6, 1.1)
Occupation (males)						
Doctors/engineers/etc.	65 (11.2)	10 (13.7)	1.0	156 (18.4)	48 (22.4)	1.0
Military (males)/housewives (females)	73 (12.6)	6 (8.2)	1.9 (0.6, 5.4)	135 (15.9)	23 (10.7)	1.3 (0.4, 3.5)
Skilled workers	400 (69.0)	48 (65.8)	1.3 (0.6, 2.6)	293 (34.5)	85 (39.7)	0.8 (0.3, 2.3)
Unskilled workers	13 (2.2)	2 (2.7)	1.0 (0.2, 5.0)	40 (4.7)	4 (1.9)	1.5 (0.5, 4.9)
Retired	29 (5.0)	7 (9.6)	0.6 (0.2, 1.8)	226 (26.6)	54 (25.2)	1.7 (0.4, 8.4)
Monthly household income (SAR)						
≤ 10,000	371 (65.4)	54 (75.0)	1.0	323 (43.1)	100 (52.9)	1.0
> 10,000	196 (34.6)	18 (25.0)	1.5 (0.9, 2.8)	426 (56.9)	89 (47.1)	*1.5 (1.1, 2.0)*
Body mass index (kg/m^2^)						
Normal (< 25)	129 (22.2)	18 (24.7)	1.0	161 (18.9)	35 (16.4)	1.0
Overweight (≥ 25–29.9)	220 (37.9)	30 (41.1)	1.0 (0.5, 1.9)	272 (32.0)	75 (35.0)	0.8 (0.5, 1.2)
Obese (≥ 30.00)	231 (39.9)	25 (34.2)	1.3 (0.7, 2.4)	417 (49.1)	104 (48.6)	0.9 (0.6, 1.3)
Current smoker						
No	*402 (69.3)*	49 (67.1)	1.0	831 (97.8)	211 (98.6)	1.0
Yes	178 (30.7)	24 (32.9)	0.9 (0.5, 1.5)	19 (2.2)	3 (1.4)	1.6 (0.5, 5.5)
Physical activity						
High/moderate	62 (10.7)	6 (8.2)	1.0	403 (47.4)	93 (43.5)	1.0
Low	518 (89.3)	67 (91.8)	0.7 (0.3, 1.8)	447 (52.6)	121 (56.5)	0.8 (0.6, 1.1)
Sun exposure/week						
Yes	181 (31.3)	20 (27.8)	1.0	103 (12.1)	27 (12.6)	1.0
No	397 (68.7)	52 (72.2)	0.8 (0.5, 1.4)	745 (87.9)	187 (87.4)	1.0 (0.7, 1.6)
Vitamin D supplements						
Yes	14 (2.4)	9 (12.3)	1.0	121 (14.2)	89 (41.6)	1.0
No	566 (97.6)	64 (87.7)	*5.7 (2.4, 13.6)*	729 (85.8)	125 (58.4)	*4.3 (3.1, 6.0)*

In Table 2, the results for the females are showing more or less similar results to that of males. The elderly females (61–75 years) are protective of developing vitamin D deficiency (0.3, 95% CI 0.1, 0.9). The females in the high-income group had 1.5 (95% CI 1.1, 2.0) times the odds for low vitamin D in comparison to normal levels. The highest percentage of females (22%) taking supplements belonged to the 44–59 years age category (*P* = 0.4). There was no significant association between vitamin D deficiency and raised blood pressure. The participants reporting low physical activity were almost equal in both categories. The odds ratio and 95% CI for physical activity, smoking, body mass index, and sun exposure were not statistically significant in the males or females. Interestingly, the average sitting time in males and females with vitamin D < 50 nmol/L was higher (9.1 ± 3.4 vs 5.9 ± 3.6) than those having vitamin D >50 nmol/L (8.4 ± 3.3 vs 5.4 ± 3.2); however, the differences were not significant.

**Fig. 1:**
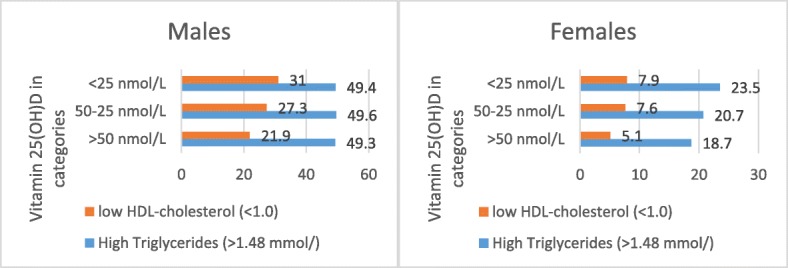
The percentage of male and female participants with dyslipidemia by vitamin D levels in Riyadh Saudi Arabia

Table 3 is showing the unadjusted odds ratio and 95% CI for the lipid profile variables with vitamin D deficiency. Males with low HDL had higher odds for developing low vitamin D [1.8 (1.1, 3.2)]. The results in females found that those with low HDL [1.4 (1.1, 2.0)] and those with high TG had higher odds for [2.8 (1.1, 7.2)] for low vitamin D. Figure 1 is showing the increasing percentage of participants with low HDL and high TG across the vitamin D categories decreasing from > 50 nmol/L, 50–25 nmol/L, and < 25 nmol/L. Although the differences were not statistically significantly different, however, the increase in high TG was obvious in females and low HDL in the males (Fig. 1). The association for total cholesterol and LDL cholesterol with vitamin D deficiency was not statistically significant.

**Table 3:** Bivariate analysis showing unadjusted odds ratio and 95% CI between lipid profile and low vitamin D in Saudi male and female participants in Riyadh, Saudi Arabia

	Males (*N* = 653)			Females (*N* = 1064)		
Variable	25(OH)D < 50 nmol/L (*n* = 580)	25(OH)D ≥ 50 nmol/L (*n* = 73)	OR (95% CI)	25(OH)D < 50 nmol/L (*n* = 850)	25(OH)D ≥ 50 nmol/L (*n* = 214)	OR (95% CI)
HDL cholesterol (mmol/L)						
Normal	373 (64.3)	56 (76.7)	*1.0*	540 (63.6)	153 (71.5)	1.0
Low (M < 1.03, F < 1.29)	207 (35.7)	17 (23.3)	*1.8 (1.1, 3.2)*	309 (36.4)	61 (28.5)	*1.4 (1.1, 2.0)*
Triglycerides (mmol/L)						
Normal	312 (53.8)	37 (50.7)	1.0	796 (93.6)	208 (97.7)	*1.0*
High (M > 1.48, F > 2.29)	268 (46.2)	36 (49.3)	0.9 (0.5, 1.4)	54 (6.4)	6 (2.3)	*2.8 (1.1, 7.2)*
LDL cholesterol (mmol/L)						
Normal	355 (61.2)	41 (56.2)	1.0	602 (70.8)	140 (65.4)	1.0
High (> 3.36)	225 (28.8)	32 (43.8)	0.8 (0.5, 1.3)	248 (29.2)	74 (34.6)	0.8 (0.6, 1.1)
Total cholesterol (mmol/L)						
Normal	347 (59.8)	40 (54.8)	1.0	542 (63.8)	120 (56.1)	1.0
High (> 5.2)	233 (40.2)	33 (45.2)	0.8 (0.5, 1.3)	308 (36.2)	94 (43.9)	0.7 (0.5, 1.0)

*p* < 0.05

Pearson correlation found that HDL was significantly negatively correlated with BMI, and this relationship was stronger in females than males (males *r* = − 0.1, *P* < 0.01; females *r* = − 0.3, *P* < 0.01). In addition, both HDL and TG though weak, but were significantly correlated with systolic blood pressure in males (HDL *r* = − 0.08, *P* = 0.03; TG *r* = 0.1, *P* < 0.01), whereas, in females, vitamin D (*r* = − 0.1, *P* < 0.01) and TG (*r* = 0.1, *P* < 0.01) were significantly correlated with systolic blood pressure.

Table 4 is showing the multivariate adjusted odds ratio with 95% CI for male and female participants. The adjusted risk of low HDL cholesterol levels in association with 25(OH)D deficiency was 2.1 times higher in males (OR_*A*_ = 2.1; 95% CI = 1.1, 3.9) and 1.3 times higher in females (OR_*A*_ = 1.3; 95% CI = 0.9, 1.9) (Table 4). A significant excess odds of having high levels of triglycerides (with a cutoff of 2.2 mmol/L) in association with 25(OH)D deficiency was observed in females (OR_*A*_ = 3.0; 95% CI = 1.1, 7.8), while this association was not observed in males (the OR_*A*_ = 0.7 mmol/L was 1.2, 95% CI = 0.4, 1.2). No significant association was observed for high total cholesterol levels or high LDL cholesterol in association with 25(OH)D in either males or females (Table 4). All potential confounders such as age, physical activity, obesity, and smoking (in males only) were adjusted while performing multivariate regression analysis.

**Table 4:** Multivariate model showing the independent association of lipid panels with standardized vitamin 25(OH)D levels in Saudi males and females in Riyadh, Saudi Arabia

Variables	Males* OR_*A*_ (95% CI)	Females** OR _*A*_ (95% CI)
Low HDL cholesterol (M < 1.03) (F < 1.29) mmol/L	*2.1 (1.1, 3.9)*	1.3 (0.9, 1.9)
High triglyceride (> 1.48 mmol/L)	0.7 (0.4, 1.2)	*3.0 (1.1, 7.9)*
High LDL cholesterol (> 3.36 mmol/L)	0.8 (0.5, 1.4)	0.8 (0.5, 1.1)

*OR*_***A***_ adjusted odds ratio

*The model was adjusted for age, body mass index, physical activity, and smoking

**The model was adjusted for age, body mass index, and physical activity

## Discussion

The impact of low vitamin D on human health is becoming increasingly significant [2]. This study found a strong association between low vitamin D and dyslipidemia, with certain differences between males and females [31]. These results support previous cross-sectional [13, 31, 32] and prospective cohort studies [33, 34] that have observed a significant association between low vitamin D and dyslipidemia [33–37]. Traditionally, vitamin D deficiency was considered to be a problem for post-menopausal women only [38]. However, the high prevalence (89% males vs 80% females), especially among the young Saudi adults negate the above notion. As is evident from the results, the percentage of participants reporting cardiovascular risk factors such as physical activity, obesity, and smoking in the vitamin D deficiency category were high than those with normal vitamin D levels. This trend emphasizes the importance of addressing low vitamin D problem equally in both males and females even though the association between the abovementioned risk factors and low vitamin D was weak but significant. In accordance with the results reported by Wang et al. [33], the results of this study found that low HDL was significantly associated with low vitamin D in the Saudi male participants, after adjusting for age, BMI, smoking, and physical activity. HDL cholesterol, which is considered the good cholesterol, has been found to be high among people who are physically active, sun-exposed, and non-smokers [39]. Although 30% of the males were exposed to the sun in comparison to 12% of the females, a greater proportion of males were vitamin D deficient. This may be because of short duration or inappropriate timings for sun exposure. A study by Sharani et al., Riyadh, Saudi Arabia, found that the optimum time to get sun exposure for vitamin D during was from 9:00 am to 10:30 am during the summer (also from 2:00 pm to 3.00 pm) and from 10:00 am to 2.00 pm during winter [40]. Another explanation could be that although not statistically significantly different, a notable percentage of females [19.7% (210)] were taking vitamin D supplements and most of them belonged to the perimenopausal age group (45 to 59 years). Therefore, we support the recommendation by the Ministry of Health to prescribe vitamin D supplements for all females after the age of 40 years. However, we recommend that supplements should be prescribed for the young Saudi adult males as well [3].

Controlling high triglyceride levels is important, especially in females, because high TG levels are not only associated with coronary artery disease but also prone to become deranged during conditions such as pregnancy, hormonal changes, and weight gain [41]. The findings in Fig. 1 relate the association between low vitamin D and dyslipidemia (*P* = 0.3) to the dose–response gradient, as the highest percentage belonged to the very low vitamin D category (< 25 nmmol/L); however, this association was not significant and future studies are required. The bivariate analysis in females did not find a significant association between low vitamin D and TG levels between 1.4 and 2.1 mmol/L; instead, we did observe a positive significant association at TG levels of 2.2 mmol/L [42]. In females, the association between HDL and vitamin D deficiency, which was significant on univariate analysis, became insignificant after adjusting for vitamin D supplementation (around 20% (*n* = 207) of the females were taking vitamin D supplements). Unhealthy lifestyle, with lack of PA and increased screening time, is also associated with low vitamin D intake [43].

Different theories have been proposed to explain how low vitamin D levels may lead to increased cholesterol levels. Researchers have suggested that calcium absorption is a factor that links the two conditions: specifically, they have proposed that increased calcium absorption may reduce the synthesis and secretion of TG in the liver [44]. Therefore, inadequate vitamin D levels may stimulate intestinal calcium uptake and consequently inhibit TG synthesis and secretion. In another theory, an alternative role for calcium was proposed whereby insoluble calcium–fatty acid complexes are formed and consequently inhibit the intestinal absorption of fatty acids. Reduced absorption of saturated fatty acids and other fats results in lower levels of cholesterol in the serum [45]. One further route of calcium’s action is that it reduces cholesterol levels by stimulating the conversion of cholesterol to bile acids. Some researchers have noted a relationship between parathyroid hormone (PTH), TG, and vitamin D in which high levels of PTH are associated with high TG and low vitamin D levels. Higher levels of vitamin D are acknowledged to reduce serum PTH [33], and this mechanism may facilitate the effect of vitamin D on TG levels. Furthermore, there is a robust body of evidence that indicates that lack of vitamin D has a corresponding impact on beta-cell function, which leads to insulin resistance, disruption of lipoprotein metabolism, and ultimately, increased TG and decreased HDL cholesterol levels [46]. In addition, vitamin D may have a direct influence on lipid metabolism and is known to play a role in the synthesis of bile acids in the liver. Hence, it is most likely that multiple mechanisms act simultaneously which results in a significant association between vitamin D deficiency and dyslipidemia.

In addition, there are some alternative explanations for the high TG or low HDL causing decreased vitamin D levels. There is enough evidence that high TG levels are associated with obesity [47]. Obese people are found to be deficient in vitamin D and it is suggested that due to the sequestering effect of a high quantity of subcutaneous fat, there is reduction in the circulating 25 (OH) vitamin D levels [48]. Another explanation can be that dyslipidemia induces high blood sugar levels that in turn lead to low vitamin D. It has been observed that sugars like fructose decrease the intestinal absorption of 25 (OH) vitamin D levels available through dietary sources [49]. Performing the recommended level of physical activity can be one possible explanation for the association between HDL and vitamin D. There is evidence available that physical activity improves the HDL cholesterol, and it also leads to improvement in the vitamin D levels (especially those doing outdoor activities). Hence, we suggest that healthy lifestyle comprising of regular physical activity may not only help in improving dyslipidemia but can also help in preventing vitamin D deficiency.

Despite the fact that several studies from KSA have reported a very high prevalence of vitamin D deficiency, none of the studies has reported an association between vitamin D deficiency and dyslipidemia. One of the major strengths of our study was inclusion of standardized vitamin D readings. Several studies consider different vitamin D cutoffs due to method-related differences. Therefore, the Vitamin D Standardization program (VDSP) recommends standardizing the vitamin D readings for accuracy and international comparison [24]. The whole process of standardizing vitamin 25(OH)D was performed by following the Vitamin D External Quality Assessment Scheme (DEQAS) laboratory-based standard protocol [24]. This was done to minimize any variations in vitamin D level measurements introduced by differences in the laboratory methods. This study followed a population-based design, wherein participants from different social classes and ethnicities were included. Hence, the results of this study can be generalized to the rest of the Saudi population. In addition, the data collectors were trained to conduct the interviews in a non-judgmental, unbiased, and objective manner. However, some element of misinformation cannot be ruled out.

However, the study had certain limitations, such as the collection of non-fasting blood samples. Blood samples were collected throughout the daytime, and it was not possible for participants to maintain a fasting state for such a long time. The variation in time between the last meal and the collection of blood samples could not be determined because the sample comprised of working and non-working participants. However, our sample comprised of healthy individuals, and the collection of non-fasting blood samples for measuring lipids is the recommended protocol for such a sample [50]. The cross-sectional study design limits us in establishing any type of causality between dyslipidemia and vitamin D deficiency.

## Conclusion

Overall, the indications from the entire body of evidence support the conclusion that, among the Saudi population, vitamin D influences serum lipid profiles and that sustaining a satisfactory level of serum vitamin D can result in favorable lipid levels. We recommend that the evidence should be strengthened by conducting further longitudinal and laboratory-based interventional studies to study how vitamin D is contributing in regulating the lipid levels in the healthy population. A public health approach for rectifying vitamin D levels in the population and advocating a healthy lifestyle is warranted. This will not only promote health and prevent the development of vitamin D deficiency-associated complications but also reduce the burden on the health care system.

## Data Availability

The datasets used and/or analyzed during the current study are available from the corresponding author on reasonable request.

## Abbreviations

BMI: Body mass index
CVD: Cardiovascular diseases
HDL: High-density lipoprotein
IPAQ: International Physical Activity questionnaire
LDL: Low-density lipoprotein
OR_*A*_: Adjusted odds ratio
SPSS: Statistical Package for the Social Sciences
TG: Triglycerides
VDSP: Vitamin D Standardization program
WC: Waist circumference
WISHES: Women In Saudi Arabia Health Examination survey
